# Thoughts Regarding the Situation of the Pediatric Cardiovascular Surgery in Brazil

**DOI:** 10.5935/1678-9741.20160039

**Published:** 2016

**Authors:** Ulisses A. Croti, Domingo M. Braile

**Affiliations:** 1 Serviço de Cardiologia e Cirurgia Cardiovascular Pediátrica de São José do Rio Preto - Hospital da Criança e Maternidade de São José do Rio Preto and Faculdade de Medicina de São José do Rio Preto (FAMERP), São José do Rio Preto, SP, Brazil.; 2 Faculdade de Medicina de São José do Rio Preto (FAMERP), São José do Rio Preto, SP, Brazil and Universidade de Campinas (UNICAMP), Campinas, SP, Brazil.

One of the authors, Dr. Ulisses A. Croti, wrote the preface for the book "Cardiopatias congênitas: rede de atenção à saúde" (Congenital Heart Disease - Health Care Network), authored by the prestigious cardiovascular surgeon Dr. Valdester C. Pinto Jr^[1]^.

The content of his work shows a Brazilian reality which should be known not only by our community, but also by authorities responsible for health course in our country.

The Brazilian Constitution of 1988 guarantees fundamental rights to all living population in Brazil, not only for Brazilians, but also for all inhabitants of this country.

Article 196 is very clear and is worthwhile to quote it in full: "Health is everyone's right and State duty, guaranteed through social and economic policies, aimed at reducing the risk of disease and other health problems, and universal and equal access to actions and services for its promotion, protection and recovery."

This article was regulated by Law No. 8,080, in September 19^th^, 1990, in its Article 2 and paragraphs, reinforced by Law Nº. 12,864, of 2013: "Health is a fundamental human right, so the State should provide the necessary conditions for its full achievement".

Based on the preface's highlighted evidences, seeking to spread the concepts and distress felt by the author, we decided to present in this Editorial a compilation based on the ideas that permeated the text.

Very responsibly I accepted the invitation to open the contents of the book "Congenital Heart Disease - Health Care Network".

My task was to present a cardiovascular surgeon's point of view on working exclusively in the care of children with congenital heart disease in Brazil.

So, after much thinking and trying to find a less thorny path to express my feelings, I concluded having the duty to be as honest as possible with myself and with children with heart disease in our country, although some might criticize and/or not agree with my ideas.

Without prejudice I begin saying that the best word to define a responsible and conscientious doctor who works in the care of children with congenital heart disease in Brazil is: distress.

Every day we live distressed with situations beyond our control and often preventing us from practicing medicine learnt in our long and difficult training as expert healthcare professionals in congenital heart disease.

It is necessary and required that the population and especially the Government at all levels understand as this been a highly complexity activity, as it is recognized in the law itself. This system's intrinsic feature implies skilled human resources, adequate infrastructure and, above all, sufficient funding for these three pillars, orchestrated by proper management, able to harmonize allowing treatment of children with congenital heart disease who can grow, study, work, form families, have children, and why not, pay taxes, so that the nation can continue to develop and promote opportunities for future generations^[2]^.

## READ ARTICLE ON PAGE 256

Such results can only be achieved if Universities and our expert Societies continue to invest heavily in improvement of population's quality education, and if the Hospitals responsible for care are better structured, in sufficient numbers and evenly distributed throughout the country to meet the demand.

To achieve these goals it is essential to have adequate economical support by Federal, State and Municipal funding, relying also on imperative support of local community, and possibly public-private partnerships, something unusual in our current management model, deserving of daily thought and discussion by all.

It is important to mention that the majority of children with heart disease in our "Educator Homeland" are covered by the Unified Health System (Sistema Único de Saúde - SUS).

Practically every day we receive phone calls from colleagues who diagnosed a newborn or an older child with congenital heart disease in the city of origin and cannot refer them to specialized treatment due to lack of vacant hospital beds.

Many times, when for a personal effort there is a vacant hospital bed, the child is transported improperly, arriving dead or with sequelae that will never be reversed at our hospital.

The State of São Paulo, where I work, made huge endeavors to even these problems implementing the Central Regulatory Health Services Offer (Central de Regulação de Ofertas de Serviços de Saúde - CROSS).

The Department of Health identified not been able to reduce infant mortality for many years, and this fact being directly dependent of improved results of paid-treatment for children with congenital heart disease. Frequently we join in this fight offering priority treatment to the sickest children born in cities from our state and also of others, representing one more burden to São Paulo State finances.

Commonly, we are faced with delayed, incomplete or even incorrect diagnosis, leading to incorrect use of antibiotics and other important medications in maintaining these children alive. Added to these difficulties is the lack of tertiary hospital vacant beds and, when available, the lack of adequate transportation.

We become increasingly distressed and aware that the service network is faulty and unable to provide proper care to children with heart disease.

It is different when looking at the national contexture panorama... worse in comparison to what happens in São Paulo, where the difficulties are even greater for the most part, especially in North and Northeast States, as already published several times in scientific journals.

The number of centers specialized and dedicated to treatment of complex congenital heart disease newborns is small, inadequate, poorly structured and underfunded.

The institutions treating the most severe cases are punished for denoting higher expenses than revenues and thus, most often prefer to not care for complex newborns or, when they do, it is poorly and inadequately done from a human or structural resource point of view.

The leaders know that if they necessary money spent, will not be endured by the system and their hospitals will be forced to close.

The newborns' situation is very serious! Many die in hospitals where they were born awaiting to be transferred, when they actually should have been born in a place where they could be treated and operated on.

Fetal echocardiography is not a routine for pregnant women in our country, this way, children who are born without adequate assistance, are transported inadequately and arrive at the specialized centers in poor medical conditions and usually infected... all over again!

These adverse conditions increase these children' risk of living by prolonging the length of hospital stay and financial expenses without any positive results, since many end up dying and leaving behind distressed and helpless involved professionals, along with the families' desperation.

Few of us work in hospitals able to receive children from less favored States, under guidance and deliberation of the National Center of High Complexity Regulation (Central Nacional de Regulação de Alta Complexidade - CNRAC). Poor children^[3]^!

## READ ARTICLE ON PAGE 219

Most often, hospitals present children with complex heart diseases that have lost the best time to provide excellent results and will pay the price living with lung diseases and complications for the rest of their lives, or won't even have this chance since they may die in the queue!

CNRAC's efforts to transfer children from less favorable States are slow and often inefficient, since it often finds obstacles in the system which does not allow them to execute its proposal correctly.

Finally, with us being fully aware of this panorama makes distress an ever-recurring present feeling, as we only get encouragement and hope when we realize efforts, although rare and single, such as this work by Dr. Valdester of utmost importance highlighting the need for even bigger and united efforts, for only then we can make the care of children with congenital heart disease better, more sustainable and ultimately worthy in Brazil^[2]^.

## References

[1] VC Pinto, Valdester C. Pinto (2015). Cardiopatias congênitas: rede de atenção à
saúde.

[2] Pinto VC, Cavalcante RC, Branco KMPC, Bezerra CTM, Maia ICL, Souza NMG (2016). Proposal of an integrated health care network system for patients
with congenital heart defects. Braz J Cardiovasc Surg.

[3] Cavalcante CTMB, Souza NMG, Pinto VC, Branco KMPC, Pompeu RG, Teles ACO (2016). Analysis of surgical mortality for congenital heart defects using
RACHS-1 risk score in a Brazilian single center. Braz J Cardiovasc Surg.

